# Supplementary material for: Non-lytic clearance of influenza B virus from infected cells preserves epithelial barrier function
Source: Nat Commun. 2019 Feb 15;10:779. doi: 10.1038/s41467-019-08617-z (PMC6377627; doi:10.1038/s41467-019-08617-z)
Supplement: Supplementary file 1 — Supplementary Information [file 41467_2019_8617_MOESM1_ESM.docx]

**Supplementary Figures and Tables**

**Supplementary Figure 1.**

**
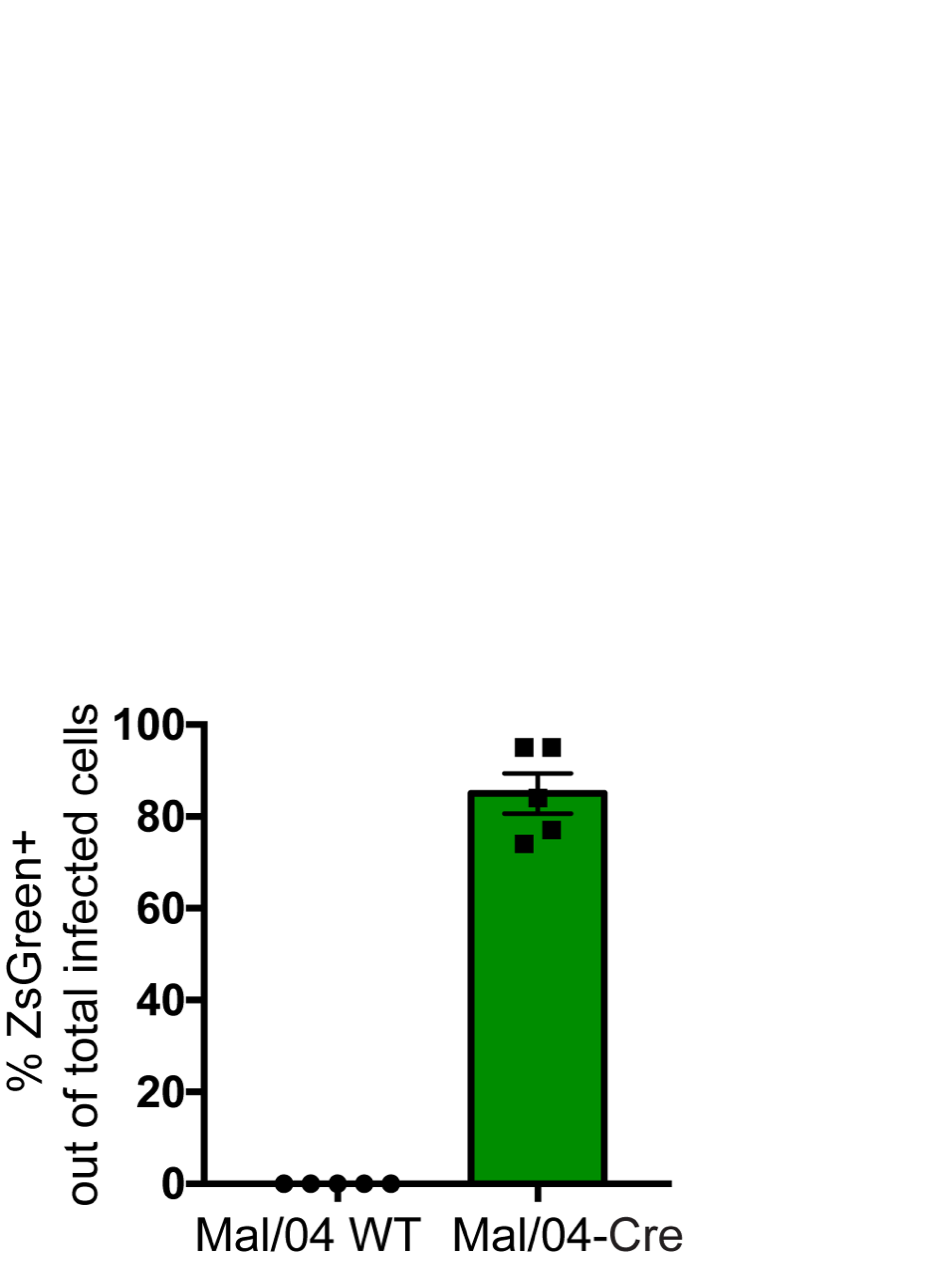
**

**Supplementary Figure 1: Mal/04-Cre activates the zsGreen reporter construct.** Microscopy of Mal/04 WT and Mal/04-Cre infection (MOI 10) of the human lung epithelial cell line, A549, harboring a floxed zsGreen fluorescent marker. At 72 hours post-infection, cells were stained with viral protein reactive serum. Infected cells (as determined by antibody staining) were scored for zsGreen reporter expression, n=5 images per sample.

**Supplementary Figure 2.**

**
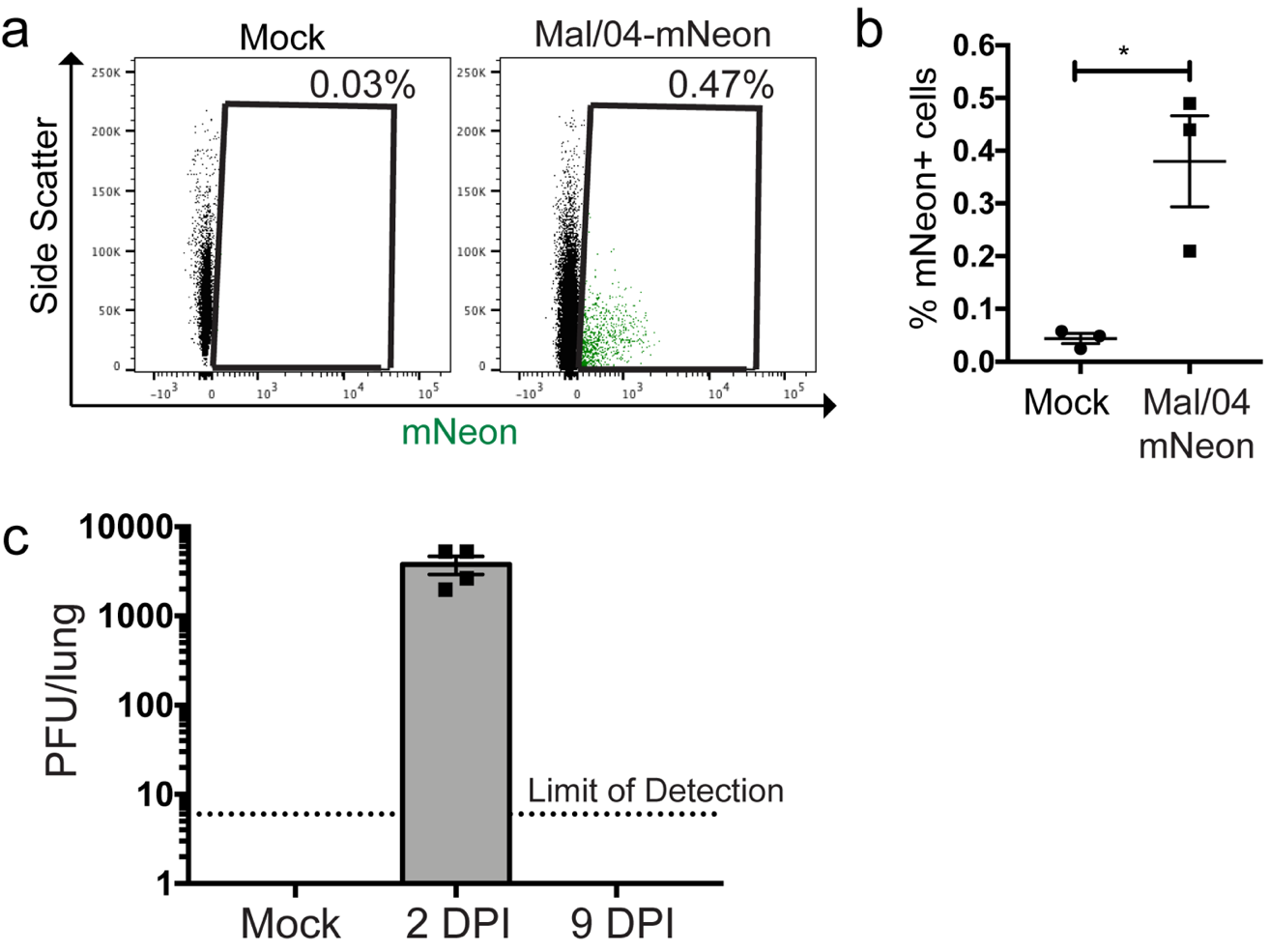
**

**Supplementary Figure 2: Mal/04 infects the murine lung epithelium and is cleared by 9 days post-infection.** A) Flow cytometry of 2DPI mNeon+ cells (infected) from the lungs of Mock and Mal/04-mNeon infected mice. B) Quantification of mNeon+ infected cells in Mock and Mal/04-Cre-infected mice. n=3 mice per sample, Student’s T-test. C) Lung homogenates were collected from were isolated from Mock and Mal/04-Cre infected murine lungs at 2 DPI and 9 DPI then plaque assays were performed to quantify infectious virus. The limit of detection was 6 plaque forming units (PFU), n= 3, 4 and 3 mice per group respectively. S.E.M was plotted to indicate variability around the mean for tested samples, *=p≤0.05, **=p≤0.001, ns=not significant.

**Supplementary Figure 3.**

**
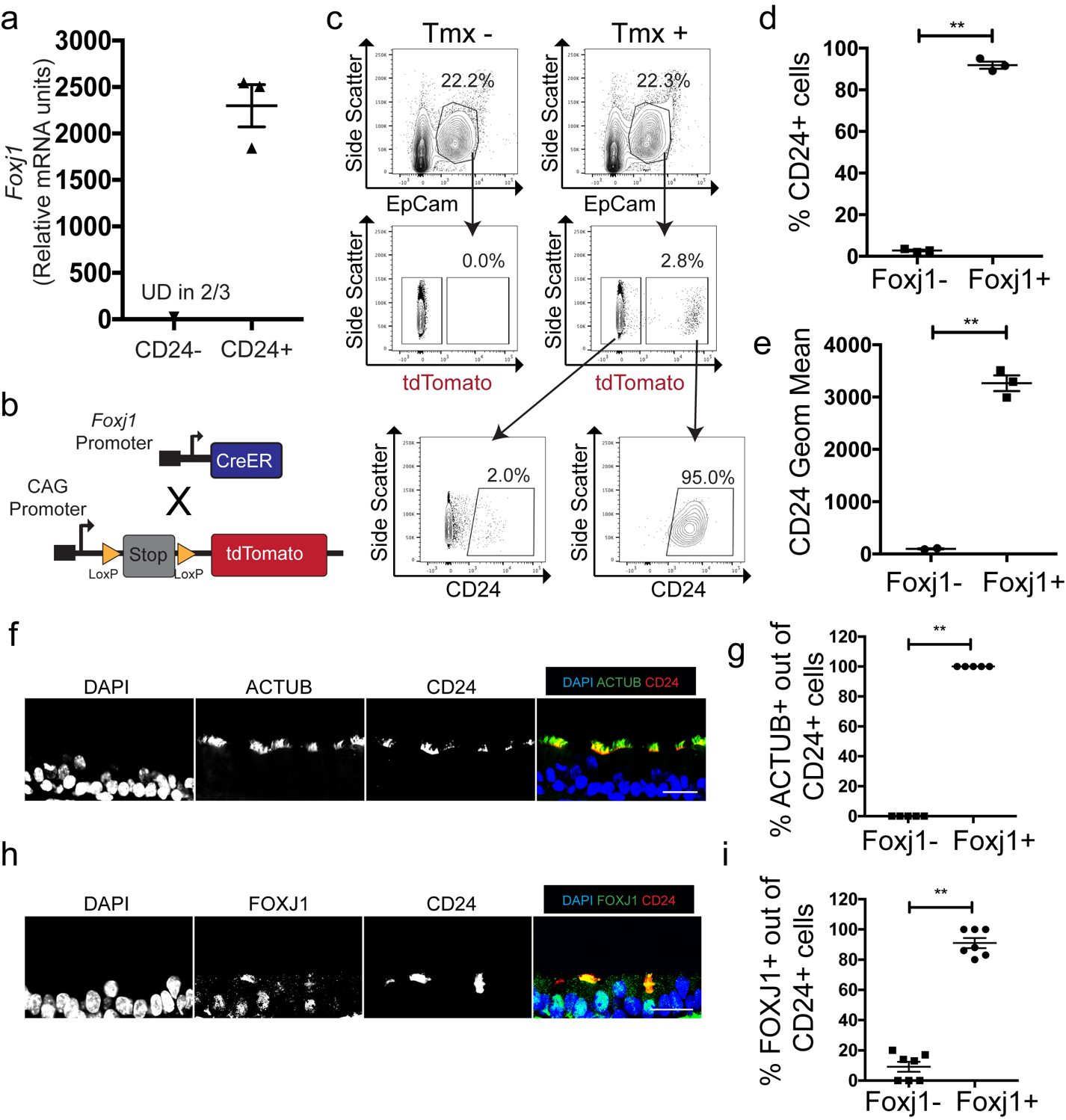
**

**Supplementary Figure 3: Validation of CD24 as an appropriate and specific marker for tracheal ciliated cells.** A) Quantitative PCR of *Foxj1* gene expression in murine epithelial cells sorted for the expression of CD24. n=3 mice per sample. B) Schematic of *foxj1*^CreERt2^; *tdTomato* transgenic mice used to genetically label Foxj1+ ciliated cells. C) Flow cytometry of epithelial cells isolated from the lungs of *foxj1*^CreERt2^; *tdTomato* mice. These mice were treated with tamoxifen to activate tdTomato expression selectively in Foxj1+ ciliated cells then stained for surface expression of CD24. D) Quantification of percentage of cells that were CD24+ in Foxj1+ and Foxj1- genetically labeled populations. n=3 mice per sample, Student’s T-test. E) Quantification of the geometric mean of CD24+ staining per cell in Foxj1+ and Foxj1- genetically labeled populations. n=3 mice per sample, Student’s T-test. F) Microscopy of cross-sectioned murine tracheas from mock infected mice where epithelial cells were stained for CD24 and acetylated tubulin for mature cilia. Scale bars = 20 μm. G) Quantification of cells that are double positive for CD24 and ACTUB, demonstrating specificity of CD24 as a marker of ciliated cells among tracheal epithelial cells. n=5 images, Student’s T-test. H) Microscopy of cross-sectioned murine tracheas from mock infected mice where epithelial cells were stained for CD24 and FOXJ1 for bonafide ciliated cells. Scale bars = 20 μm. I) Quantification of cells that are double positive for CD24 and FOXJ1, demonstrating specificity of CD24 as a marker of ciliated cells among tracheal epithelial cells. n=7 images, Student’s T-test. S.E.M was plotted to indicate variability around the mean for tested samples, *=p≤0.05, **=p≤0.001, ns=not significant.

**Supplementary Figure 4.**

**
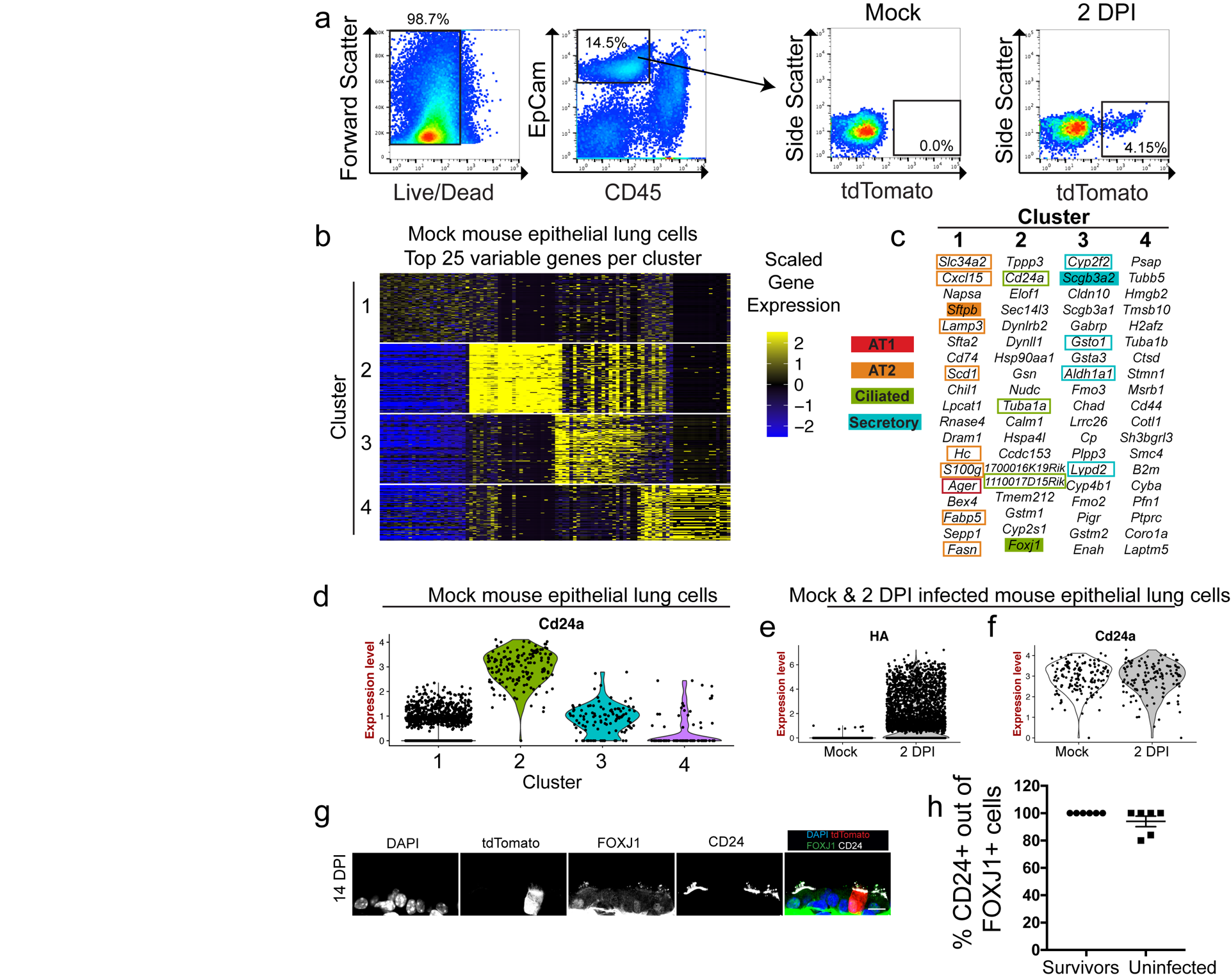
**

**Supplementary Figure 4: Validation of CD24 as a marker that is maintained in ciliated cells infected with Mal/04-Cre virus.** A) FACS gating strategy to isolate live EpCam+ CD45- epithelial lung cells from Mock and Mal/04-Cre infected *lox-STOP-lox-tdTomato* transgenic mice at 2 DPI. B) Heatmap demonstrating standard gene expression profile of lung epithelial cells from the lungs of mock infected mice. Top 25 variable genes for each cluster (cluster markers) shown for representative 100 cells per cluster. Gene expression is scaled, where relative expression of each gene is in comparison to all other cells. C) Top 20 cluster markers for each cluster with genes associated with canonical cell types (alveolar type I, alveolar type II, ciliated, secretory) indicated in the legend. Solid boxes indicated canonical cell markers and open boxes indicate genes correlated with cell types by previously published single cell RNA-seq datasets^51^. D) *Cd24a* gene expression in mock sample cells across all clusters. E) Viral hemagglutinin (*HA)* gene expression in mock and 2DPI samples. F) *Cd24a* gene expression in mock and 2DPI samples. All expression is normalized to the total reads per cell, scaled to 10,000 reads total and log-normalized, according to Seurat’s default settings. G) Microscopy of cross-sectioned murine tracheas from the lungs of mice 14 days post-infection where epithelial cells were stained for CD24 and FOXJ1 for bonafide ciliated cells. Scale bars = 10 μm. H) Quantification of tdTomato- (uninfected cells), and tdTomato+ (survivor cells) that are double positive for CD24 and FOXJ1. n=6 images.

**Supplementary Figure 5.**

**
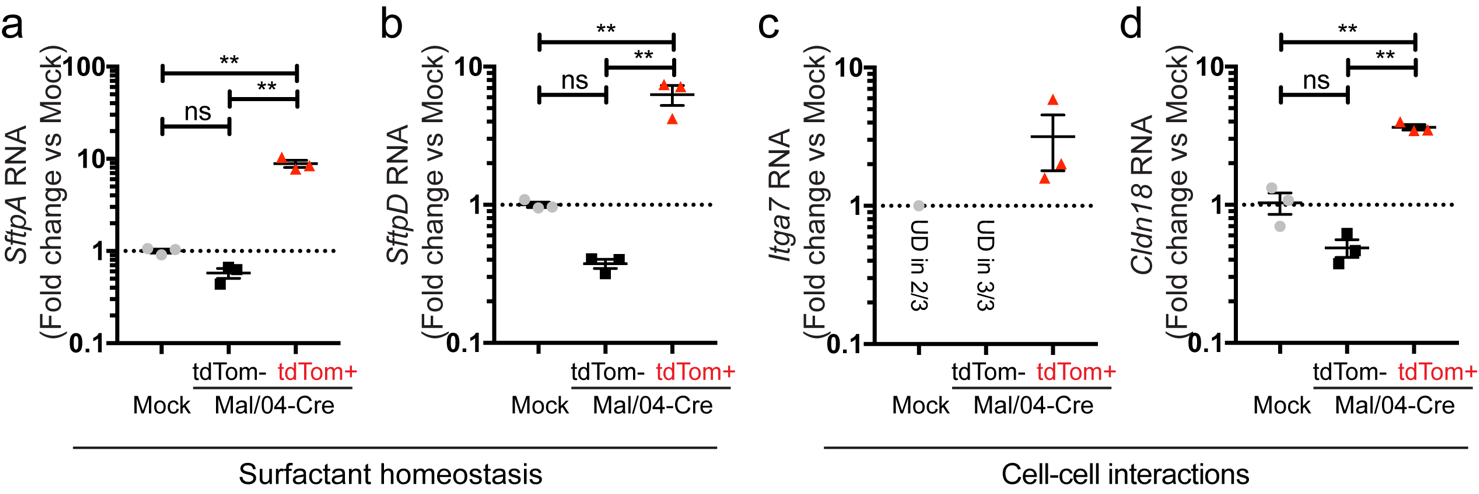
**

**Supplementary Figure 5: Survivor ciliated-like cells uniquely upregulate surfactants and cytoskeletal genes.** A) Quantitative PCR measuring RNA levels of gene of interest in flow sorted EpCam+CD24+ mock infected cells, tdTomato- (uninfected cells) and tdTomato+ (survivor cells) at 14 days post-infection. A) *Surfactant Protein A (SftpA)* B) *Surfactant Protein D (SftpD)* C) *Integrin 7 (Itga7)* D) *Claudin 18 (Cldn18)*. n= 3 mice per sample, one-way ANOVA. S.E.M was plotted to indicate variability around the mean for tested samples, *=p≤0.05, **=p≤0.001, ns=not significant.

**Supplementary Figure 6.**

**
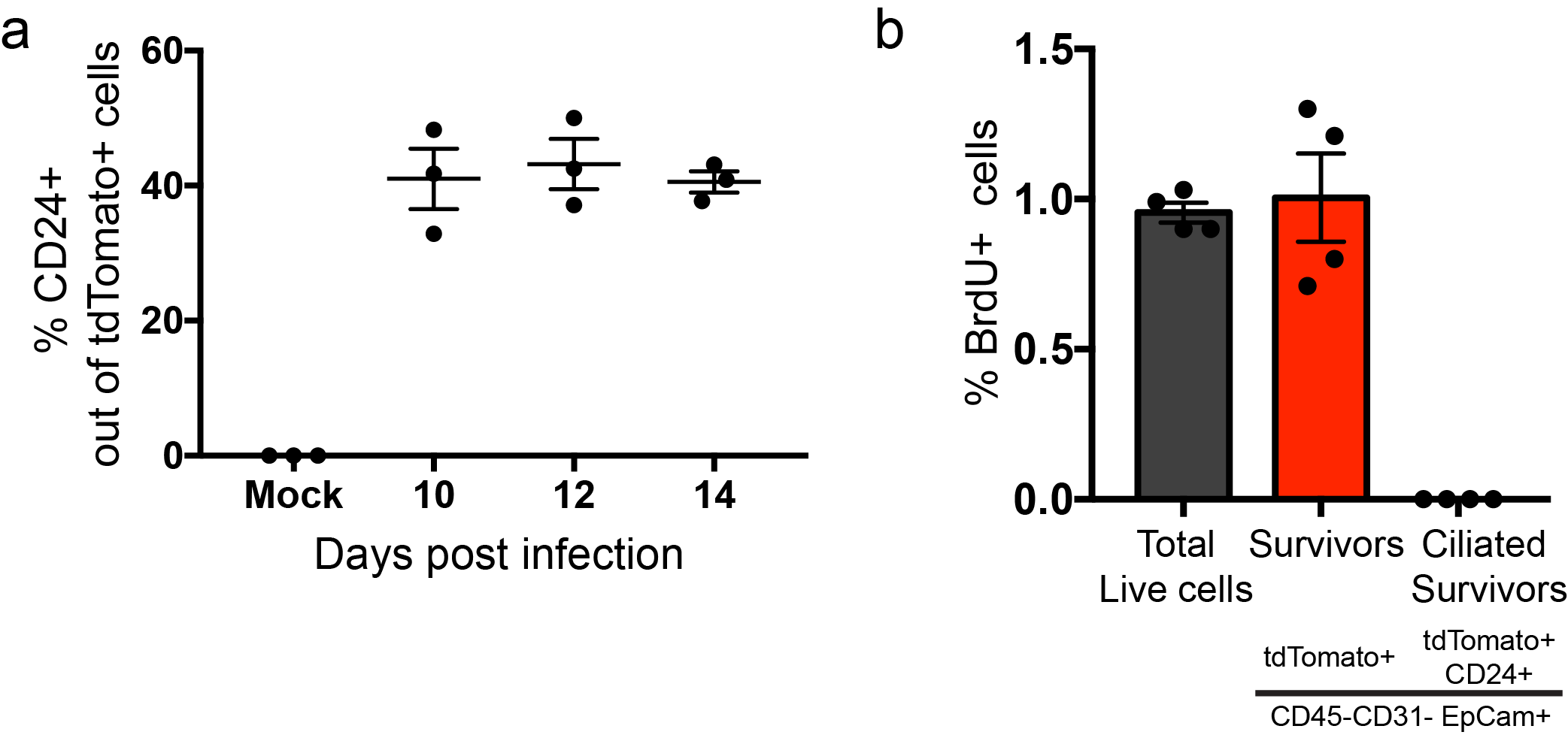
**

**Supplementary Figure 6: The percentage of CD24+ ciliated-like cells in the survivor cell population is stable over time and is not labeled by BrdU staining.** A) Flow cytometry of Epcam+CD24+ tdTomato+ cells (survivor ciliated cells) from the lungs of Mock and Mal/04-Cre infected mice at 10-14 days post-infection. n=3 mice per timepoint. B) Mal/04-Cre infected mice were treated with BrdU at 10-14 days post-infection to label proliferating cells. Flow cytometry was performed on total live cells, CD45-CD31-EpCam+tdTomato+ survivor epithelial cells, and CD45-CD31-EpCam+tdTomato+ CD24+ survivor ciliated cells from the lungs of animals collected at 14DPI. n= 4 mice per sample.

**Supplementary Figure 7.**


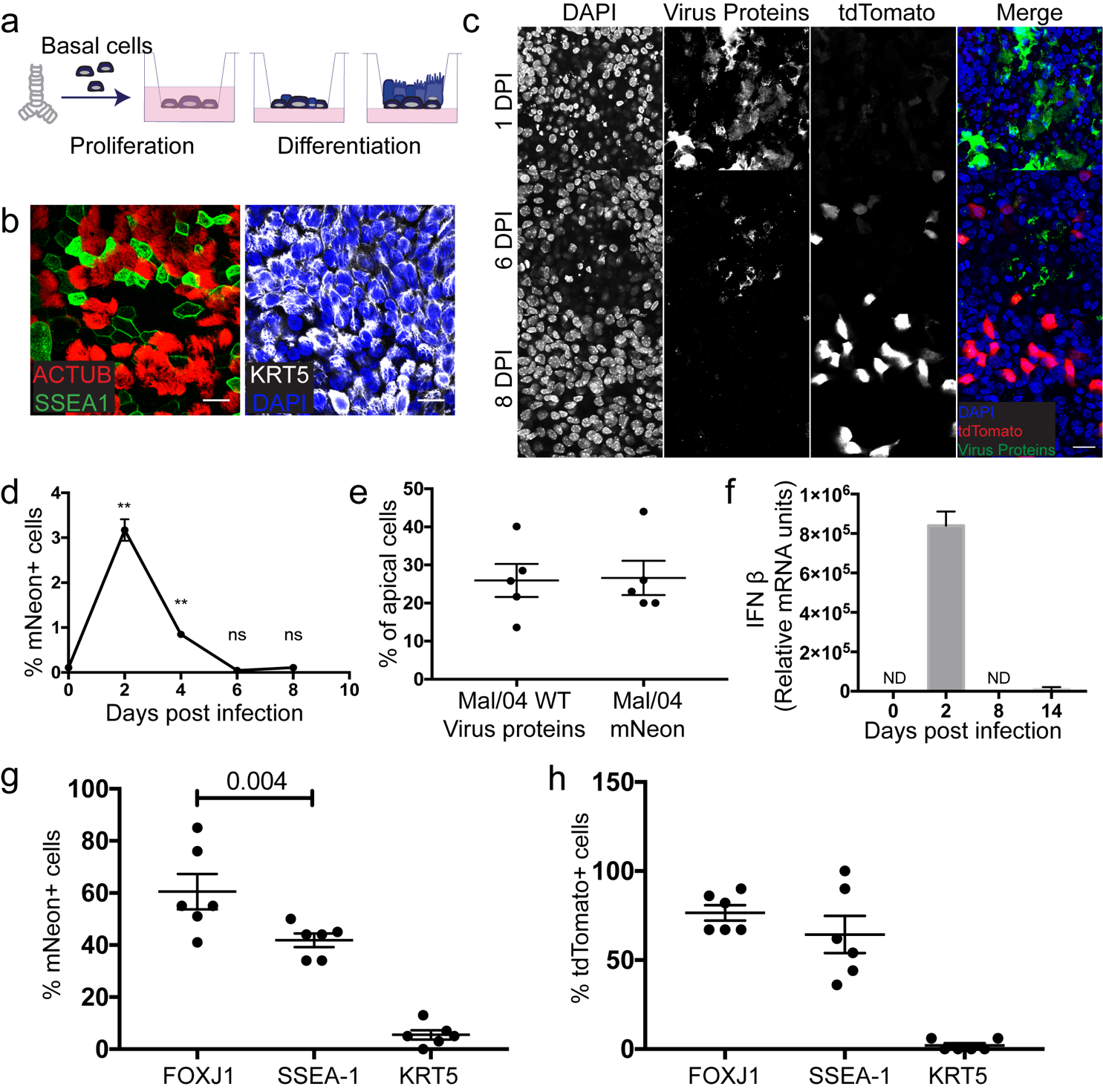


**Supplementary Figure 7: Ciliated-like cells surviving Mal/04-Cre infection can be modeled in differentiated air-liquid interface culture.** A) Murine basal cells were isolated from tracheas and grown to confluence then exposed to air for differentiation for 14-21 days. B) Whole-mount images of apical (ACTUB, SSEA1) and basolateral (KRT5, DAPI) sections of differentiated cultures. At the time of infection, there were approximately equal numbers of apical ciliated and secretory cells. Scale bars = 20 μm C) Microscopy time course of cultures isolated from *lox-stop-lox-tdTomato* transgenic mice infected with Mal/04-Cre stained for viral proteins with endogenous tdTomato marking survivor cells. Scale bars = 20 μm D) Time course of Mal/04-mNeon infected cells by flow cytometry. n=3 cultures per timepoint, one-way ANOVA. E) Quantification of apical surface area of cells infected with either Mal/04 WT and stained for viral proteins or Mal/04-mNeon. n=5 images per sample, at least 5 marker+ cells per image. F) Transcriptional analysis of IFN-β production in differentiated cultures after infection with Mal/04-Cre. n=2 cultures per timepoint, mRNA normalized to endogenous 18S control. G) Quantification of Mal/04-mNeon infected cells stained for cell-type specific markers: FOXJ1 (ciliated), SSEA-1 (secretory), KRT5 (basal). n=6 images per marker, at least 8 tdTomato+ cells per image, one-way ANOVA. H) Quantification of tdTomato+ survivor cells stained for cell-type specific markers: FOXJ1 (ciliated), SSEA-1 (secretory), KRT5 (basal). n=6 images per marker, at least 8 tdTomato+ cells per image. S.E.M was plotted to indicate variability around the mean for tested samples, *=p≤0.05, **=p≤0.001, ns=not significant.

**Supplementary Figure 8.**

**
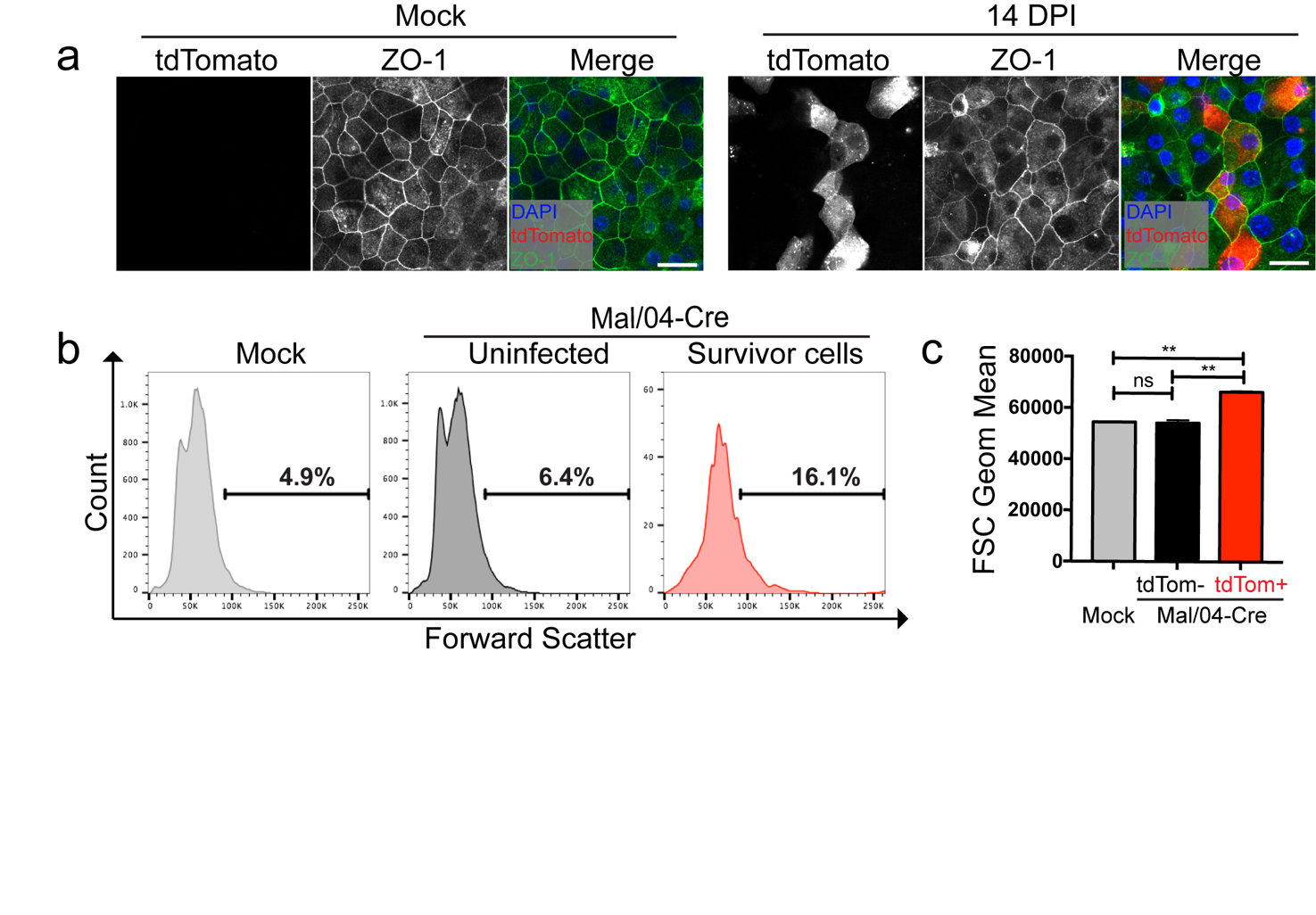
Supplementary Figure 8: Morphological changes to survivor cells can be modeled *in vitro***. A) Differentiated cultures of cells isolated from *lox-STOP-lox-tdTomato* transgenic mice were infected with Mal/04-Cre and stained for ZO-1 with tdTomato marking survivor cells. Scale bars = 20 μm. B) Cells were isolated from mock and Mal/04-Cre infected cultures and forward scatter was measured for mock infected cells, tdTomato^-^ (uninfected cells) and tdTomato+ (survivor cells) C) Quantification of the geometric mean of cell forward scatter, n=2 cultures per group, one-way ANOVA. S.E.M was plotted to indicate variability around the mean for tested samples, *=p≤0.05, **=p≤0.001, ns=not significant.

**Supplementary Figure 9.**

**
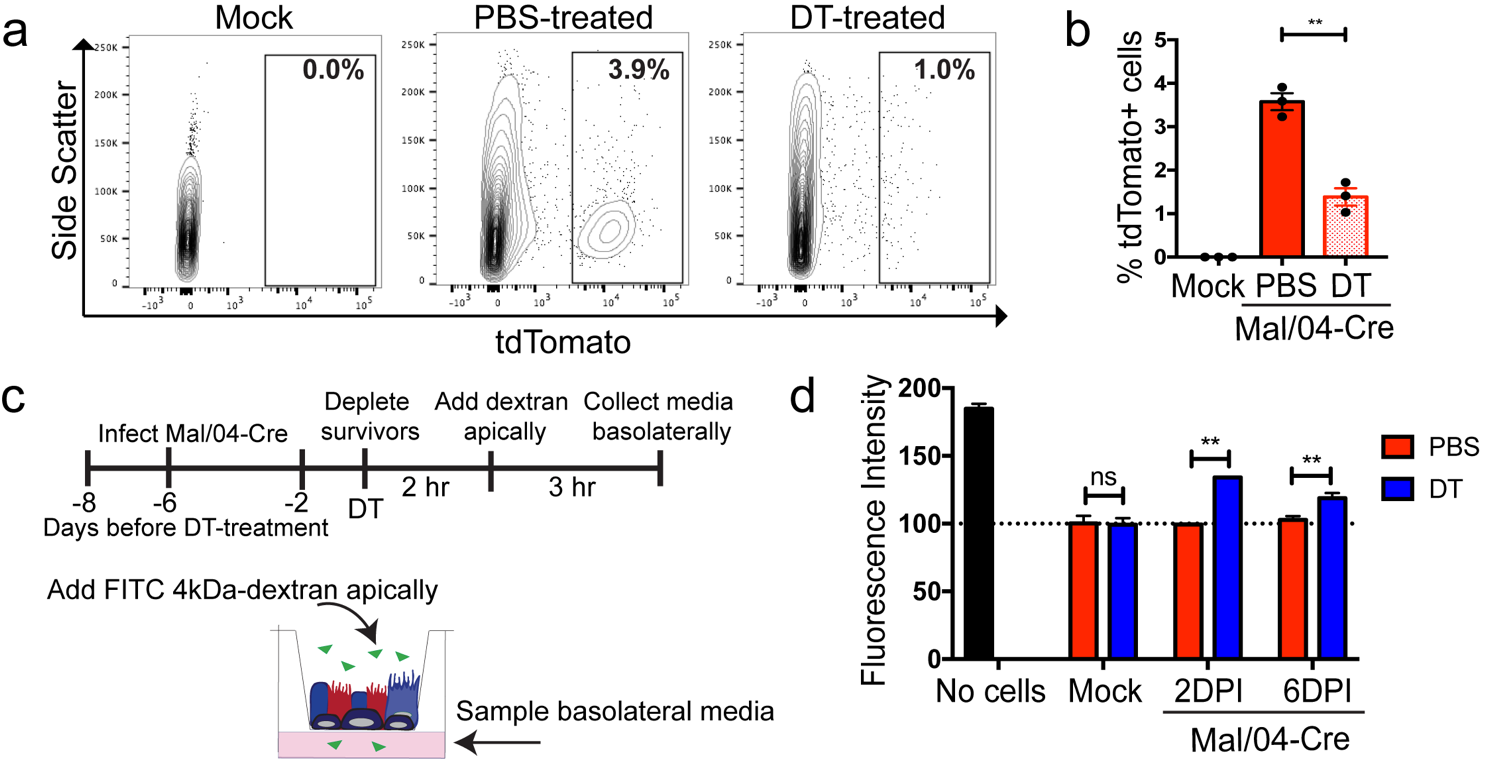
Supplementary Figure 9: Epithelial permeability from survivor cell depletion can be modeled *in vitro***. A) Differentiated cultures of cells isolated from *tdTomato;DTR* heterogenic transgenic mice were infected with Mal/04-Cre then treated with either PBS or diphtheria toxin to ablate survivor cells. tdTomato+ cells were quantified using flow cytometry. B) Quantification of the percentage of tdTomato+ cells per culture, n=3 cultures per group, one-way ANOVA. C) Schematic of permeability measurements using FITC-labeled 4kDa dextran after diphtheria toxin treatment of membranes. D) Quantifications of fluorescently labeled dextran leakage through membrane at 2 and 6 DPI of WT and *lox-STOP-lox-DTR* transgenic membranes (either mock infected with PBS or infected with Mal/04-Cre and treated with PBS or diphtheria toxin to ablate survivor cells). n=3 membranes per sample, two-way ANOVA. S.E.M was plotted to indicate variability around the mean for tested samples, *=p≤0.05, **=p≤0.001, ns=not significant.

**Supplementary Figure 10.**

**
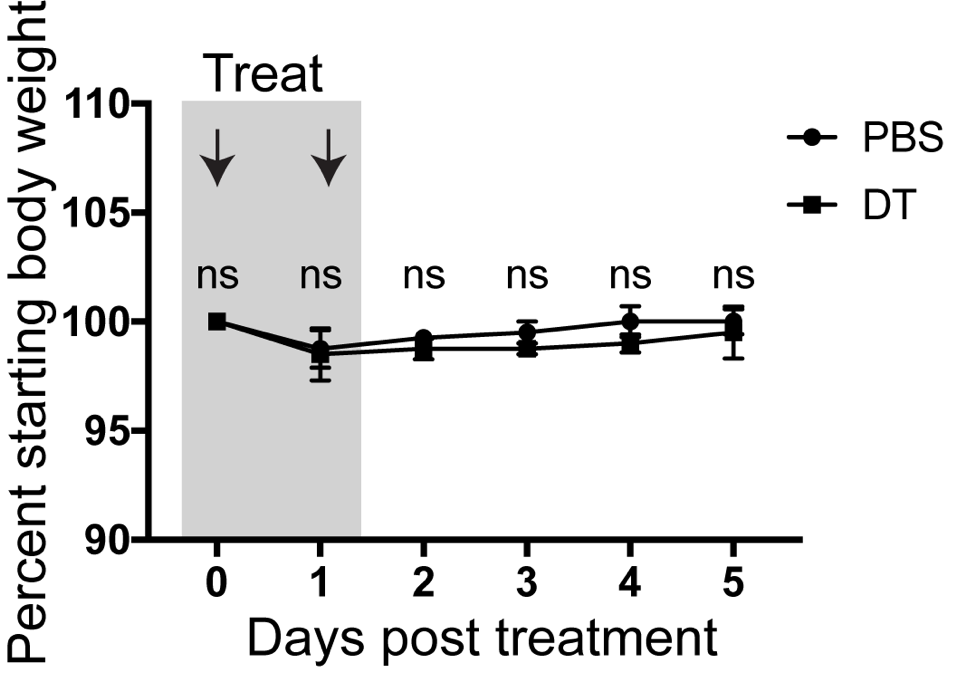
**

**Supplementary Figure 10: Diphtheria toxin treatment alone does not cause morbidity in mice**. Body weight curves of mice treated with PBS or diphtheria toxin on days 0 and 1. n= 4 mice per sample, two-way ANOVA. S.E.M was plotted to indicate variability around the mean for tested samples, *=p≤0.05, **=p≤0.001, ns=not significant.

**Supplementary Table 1: Top 200 variably expressed genes (in descending order) from bulk RNA-sequencing between Mock, 2 DPI (infected), 14 DPI (uninfected) and 14 DPI (survivor cells) as displayed in the heat map in Fig 4C.**

**Supplementary Table 2: Top 100 differentially expressed genes (in descending order) between Mock and 2 DPI infected samples from RNA-sequencing as displayed in Figure 4E.**

**Supplementary Table 3: Primer sequences for virus cloning and rescue.**

**Supplementary Table 4: Antibody profiles used for flow cytometry and microscopy assays.**
