# Supplementary material for: Non-lytic clearance of influenza B virus from infected cells preserves epithelial barrier function
Source: Nat Commun. 2019 Feb 15;10:779. doi: 10.1038/s41467-019-08617-z (PMC6377627; doi:10.1038/s41467-019-08617-z)
Supplement: Supplementary file 3 — Description of Additional Supplementary Files [file 41467_2019_8617_MOESM3_ESM.pdf]

**Description of Additional Supplementary Files**

File Name: Supplementary Data 1

Description: Gene Ontology Analysis of differentially expressed genes in survivor cells. The top 500 differentially expressed genes between 14 DPI uninfected and 14 DPI survivor cells as ranked by p value were uploaded to DAVID software for Biological Process (BP) Gene Ontology analysis. GO terms with Bonferroni adjusted $p < 0.05$ from this analysis were plotted in Fig 4F.
