# Supplementary material for: Non-lytic clearance of influenza B virus from infected cells preserves epithelial barrier function
Source: Nat Commun. 2019 Feb 15;10:779. doi: 10.1038/s41467-019-08617-z (PMC6377627; doi:10.1038/s41467-019-08617-z)
Supplement: Supplementary file 5 — Reporting Summary [file 41467_2019_8617_MOESM5_ESM.pdf]

nature research

Corresponding author(s): NCOMMS-17-33588D

Last updated by author(s): 2019-1-21

Reporting Summary

Nature Research wishes to improve the reproducibility of the work that we publish. This form provides structure for consistency and transparency in reporting. For further information on Nature Research policies, see Authors & Referees and the Editorial Policy Checklist.

Statistics

For all statistical analyses, confirm that the following items are present in the figure legend, table legend, main text, or Methods section.

n/a	Confirmed	
☐	☒	The exact sample size (n) for each experimental group/condition, given as a discrete number and unit of measurement
☐	☒	A statement on whether measurements were taken from distinct samples or whether the same sample was measured repeatedly
☐	☒	The statistical test(s) used AND whether they are one- or two-sided<br>*Only common tests should be described solely by name; describe more complex techniques in the Methods section.*
☒	☐	A description of all covariates tested
☐	☒	A description of any assumptions or corrections, such as tests of normality and adjustment for multiple comparisons
☐	☒	A full description of the statistical parameters including central tendency (e.g. means) or other basic estimates (e.g. regression coefficient) AND variation (e.g. standard deviation) or associated estimates of uncertainty (e.g. confidence intervals)
☐	☒	For null hypothesis testing, the test statistic (e.g. F, t, r) with confidence intervals, effect sizes, degrees of freedom and P value noted<br>*Give P values as exact values whenever suitable.*
☒	☐	For Bayesian analysis, information on the choice of priors and Markov chain Monte Carlo settings
☒	☐	For hierarchical and complex designs, identification of the appropriate level for tests and full reporting of outcomes
☒	☐	Estimates of effect sizes (e.g. Cohen's d, Pearson's r), indicating how they were calculated

Our web collection on statistics for biologists contains articles on many of the points above.

Software and code

Policy information about availability of computer code

Data collection	Bulk RNA-sequencing samples were analyzed on 2 lanes of Illumina HiSeq 2500 in rapid run mode. The raw and normalized data is accessible at NCBI GEO under the accession number GSE116032. The reads were mapped to the mouse transcriptome (mm10) and the B/Mal/04 genome using Bowtie, and samples were run on Basespace (Illumina).<br><br>Single cell RNA-sequencing libraries were sequenced over 2 lanes of Illumina HiSeq 4000 with 150 bp paired end reads. The raw and normalized data is accessible at NCBI GEO under the accession number GSE116032. The reads were then processed according to 10X Genomics Cell Ranger pipeline.
Data analysis	Bulk RNA-sequencing reads were then imported into DeSeq2 for analysis.<br><br>The single cell RNA-sequencing count matrix was then analyzed using R and the R package Seurat.

For manuscripts utilizing custom algorithms or software that are central to the research but not yet described in published literature, software must be made available to editors/reviewers. We strongly encourage code deposition in a community repository (e.g. GitHub). See the Nature Research guidelines for submitting code & software for further information.

Data

Policy information about availability of data

All manuscripts must include a data availability statement. This statement should provide the following information, where applicable:

- Accession codes, unique identifiers, or web links for publicly available datasets
- A list of figures that have associated raw data
- A description of any restrictions on data availability

The raw and analyzed for both single-cell RNA sequencing and bulk RNA-sequencing is accessible at NCBI GEO under the accession number GSE116032. All other data and reagents are available from the corresponding author at request.

Field-specific reporting

Please select the one below that is the best fit for your research. If you are not sure, read the appropriate sections before making your selection.

☒ Life sciences ☐ Behavioural & social sciences ☐ Ecological, evolutionary & environmental sciences

For a reference copy of the document with all sections, see nature.com/documents/nr-reporting-summary-flat.pdf

Life sciences study design

All studies must disclose on these points even when the disclosure is negative.

Sample size	All experiments included at least 3 biological replicates as indicated in the figure legend. Sample sizes were chosen based on the expected magnitude and variance of the phenotype and most experiments included 3-6 samples per treatment group.
Data exclusions	No samples were excluded unless specifically stated in the figure legend and methods.
Replication	All experiments were replicated at least twice.
Randomization	Both male and female animals were used and randomly assigned to mock and infected treatment groups.
Blinding	No blinding was performed.

Reporting for specific materials, systems and methods

We require information from authors about some types of materials, experimental systems and methods used in many studies. Here, indicate whether each material, system or method listed is relevant to your study. If you are not sure if a list item applies to your research, read the appropriate section before selecting a response.

Materials & experimental systems

n/a	Involved in the study	
☐	☒	Antibodies
☐	☒	Eukaryotic cell lines
☒	☐	Palaeontology
☐	☒	Animals and other organisms
☒	☐	Human research participants
☒	☐	Clinical data

Methods

n/a	Involved in the study	
☒	☐	ChIP-seq
☐	☒	Flow cytometry
☒	☐	MRI-based neuroimaging

Antibodies

Antibodies used	See Supplementary Table 4
Validation	See Supplementary Table 4

Eukaryotic cell lines

Policy information about cell lines

Cell line source(s)	MDCK and A549 were both purchased from ATCC.
Authentication	None of the cell lines were additionally authenticated.
Mycoplasma contamination	Cells were not monitored for mycoplasma.
Commonly misidentified lines (See ICLAC register)	*Name any commonly misidentified cell lines used in the study and provide a rationale for their use.*

Animals and other organisms

Policy information about studies involving animals; ARRIVE guidelines recommended for reporting animal research

Laboratory animals	Wild-type C57BL/6, B6.Cg-Gt(ROSA)26Sortm14(CAG-tdTomato)Hze/J (tdTomato) and C57BL/6-Gt(ROSA) 26Sortm1(HBEGG)Awai/

Laboratory animals	J (DTR) mice were purchased from The Jackson Laboratory. Foxj1CreERt2 transgenic mice were developed by Dr. Chay T. Kuo. Both male and female animals were used and randomly assigned to mock and infected treatment groups.
Wild animals	The study did not involve wild animals.
Field-collected samples	The study did not involve samples collected from the field.
Ethics oversight	All experiments involving animals were conducted in accordance with Duke University Animal Care and Use Committee.

Note that full information on the approval of the study protocol must also be provided in the manuscript.

Flow Cytometry

Plots

Confirm that:

☒ The axis labels state the marker and fluorochrome used (e.g. CD4-FITC).

☒ The axis scales are clearly visible. Include numbers along axes only for bottom left plot of group (a 'group' is an analysis of identical markers).

☒ All plots are contour plots with outliers or pseudocolor plots.

☒ A numerical value for number of cells or percentage (with statistics) is provided.

Methodology

Sample preparation	Mouse lung epithelial cells were isolated from WT, tdTomato and DTR mice for differentiated cell culture. Lungs were perfused with 5 mL PBS then inflated with 1 mL Dispase (Corning) and 1 mL 1% low-melt NuSieve agarose (Lonza) in water. Lungs were then digested at room temperature in dispase for 45 minutes, further homogenized using a razor blade in DMEM containing DNase I (Sigma-Aldrich) and filtered through a 70 [illegible]m filter (Corning). Red blood cells were removing using 1X Red blood cell lysis buffer (BD Biosciences) then washed in PBS 1% BSA before staining. See Supplementary Table 4 for antibodies used.
Instrument	Cells were analyzed on a FACSCanto II or a Fortessa X-20 (BD) machine with standard laser and filter combinations.
Software	Data was visualized and processed with FlowJo software.
Cell population abundance	Samples for bulk RNA-sequencing were sorted based on tdTomato, CD24, EpCam and CD45 as indicated in the figure legend then RNA was prepped from (Mock: 78,000, 2 DPI: 4,100, 14 DPI SC: 44,000, 14 DPI: 100,000) cells<br><br>Samples for single-cell RNA-sequencing contained 100,000 Mock cells and 24,000 2DPI cells after the sort. Cells were washed and libraries were prepped from 5000 cells using Chromium Single Cell 3′ Reagent Kit (10X Genomics)
Gating strategy	Gating strategies are provided for bulk RNA-sequencing (Fig 4) and single-cell RNA-sequencing (Supplementary Fig 4).

☒ Tick this box to confirm that a figure exemplifying the gating strategy is provided in the Supplementary Information.
